# A novel application of a data mining technique to study intersections in the social determinants of mental health among young Canadians

**DOI:** 10.1016/j.ssmph.2021.100946

**Published:** 2021-10-21

**Authors:** M.A. McIsaac, M. Reaume, S.P. Phillips, V. Michaelson, V. Steeves, C.M. Davison, A. Vafaei, N. King, W. Pickett

**Affiliations:** a School of Mathematical and Computational Sciences, University of Prince Edward Island, Charlottetown, PEI, Canada; b Faculty of Medicine, University of Ottawa, Ottawa, ON, Canada; c Centre for Studies in Primary Care, Queen's University, Kingston, ON, Canada; d Department of Health Sciences, Brock University, St. Catharines, ON, Canada; e Department of Criminology, University of Ottawa, Ottawa, ON, Canada; f Department of Public Health Sciences, Queen's University, Kingston, ON, Canada

**Keywords:** Adolescence, Epidemiology, Intersectionality, Mental health, Social determinants

## Abstract

**Objectives:**

Adolescent mental health is an emergent clinical and public health priority in Canada. Gender-based differences in mental health are well established. The objective of this study was to evaluate a new data mining technique to identify social locations of young Canadians where differences in mental health between adolescent males and females were most pronounced.

**Methods:**

We examined reports from 21,221 young Canadians aged 11–15 years (10,349 males, 10,872 females) who had responded to a 2018 national health and health behaviours survey. Using recursive partitioning for subgroup identification (SIDES), we identified social locations that were associated with the strongest differences between males and females for three reported mental health outcomes: positive psychosomatic health, symptoms of depression, and having a diagnosed mental illness.

**Results:**

The SIDES algorithm identified both established and new intersections of social factors that were associated with gender-based differences in mental health experiences, most favouring males.

**Discussion:**

This analysis represents a novel proof-of-concept to demonstrate the utility of a subgroup identification algorithm to reveal important differences in mental health experiences between adolescent males and females. The algorithm detected new social locations (i.e., where gender intersected with other characteristics) associated with poor mental health outcomes. These findings set the stage for further intersectional research, involving both quantitative and qualitative analyses, to explore how axes of discrimination may intersect to shape potential gender-based health inequalities that emerge during childhood.

## Highlights

• The mental health of adolescent males and females differ substantially.
•We provide a novel quantitative exploration of gender-based health inequalities.
•Recursive partitioning identified subgroups with largest mental health differences.
•Intersections found with gender and affluence, age, religion, parenting, background.

## 1 Introduction

During the early years of childhood, reports on the health status of males and females reflect more similarities than differences (WHO, 2015; UNICEF Office of Research, 2016). By adolescence, however, differences in reported health outcomes begin to emerge. These are especially pronounced in the area of mental health (Torsheim et al., 2006) where gender-based differences in mental health outcomes are evident among adolescents and can become entrenched into adulthood (Patton et al., 2016). This is particularly important in Canada, where mental health and mental illness have become major health burdens in both children and adults (Freeman et al., 2011). In this paper, we explore the novel application of a data mining technique to see whether gender-based mental health differences cluster around other social, biological or environmental factors.

While our method is not intended to identify or test the mechanisms or pathways underlying specific inequalities or inequities at this point, this analysis can advance intersectional research by providing proof-of-concept for a method that can identify potential social locations of interest to intersectional scholars and practitioners. These social locations can be explored through further research to examine if and how intersecting axes of discrimination contribute to poor health outcomes. Such information is vital for the development and targeting of focused interventions.

Our current analysis is therefore a first step in this larger project. Our main goal in this paper is to test the utility of a data-driven method (recursive partitioning for subgroup identification, or SIDES) (Lipkovich et al., 2011) to identify subpopulations that may occupy social locations at the intersection of various axes of discrimination and who may, as a result, have poorer mental health outcomes. SIDES was developed in the field of personalized medicine in order to identify groups of patients who experience enhanced treatment effects (Lipkovich et al., 2011). Its use is attractive because SIDES algorithmically identifies a number of (potentially overlapping) locations of significant inequality. Rather than simply adding together various effects or hypothesizing intersections with respect to predetermined drivers, the method can potentially make visible important new social locations (and new intersecting drivers) of significant inequality.

We applied a SIDES analysis to data collected as part of a longstanding Canadian adolescent health and health behaviours survey (Craig et al., 2020). Our working hypothesis was that sub-populations of adolescent males and females would report differences in indicators of mental health status, both overall and within intersecting social and cultural strata. Of course, further research is required to go beyond this first step of identifying social locations of interest, specifically to understand if and how differential power relationships interact at those locations to shape mental health.

### 1.1 Our intersectional lens

The gender analytic framework that shapes our study provides a useful lens for framing an intersectional investigation. Consideration of intersectional factors contributing to gendered differences is important because, although some health differences or *health inequalities* between individuals and or population groups are rooted in genetic or biological factors, these account for only a portion of the health disparities across populations. There are, in addition, many social and environmental health determinants such as the level of family income, parents’ level of education, social and physical environmental exposures or experiences of racial or gender privilege or discrimination that have profound health effects (Mikkonen & Raphael, 2010). Intersectionality theory suggests that the health impacts of different social or environmental factors should not be considered individually, or merely in an additive way, but instead should be seen as being located on axes of “interlocking systems of oppression” and privilege (Collins & Bilge, 2020). This theory, articulated by Kimberlé Crenshaw(Crenshaw, 1991, pp. 1241–1299), refers to the overlapping and dynamic interplay between various drivers of marginalization and/or privilege (power and domination and/or oppression). Social and environmental determinants of health intersect and act synergistically to situate individuals at various social locations that correlate with poorer or better outcomes.

While qualitative research paradigms have a longer history in their application of intersectionality, the field of social epidemiology (Bauer, 2014; Bauer & Scheim, 2019) has begun to embrace its tenets in innovative ways in order to study quantitatively the intersecting effects of demographic and other factors that lead to health inequalities, social marginalization or privilege. A fundamental analytic step in this process is the identification of social locations, both established and novel, that are associated with differences in health outcomes. The study of social locations can additionally help to highlight health *inequities* – health differences that are avoidable, unfair and systematically disadvantage or advantage particular social groups (Braveman & Gruskin, 2003). Members of particular population sub-groups may have poorer outcomes because of the ways that structural inequities (such as misogyny or racism) shape their experiences (Paradies, 2006). Contemporary analytic methods such as recursive partitioning (Zhang & Singer, 2013), multilevel analysis of individual heterogeneity and discriminatory accuracy (MAIHDA) (Merlo, 2018) and causal mediation analyses (Pearl, 2009) offer new promise for understanding different health experiences.

## 2 Material and methods

### 2.1 Data source

We analyzed recent (2017-18) Canadian data from an adolescent health study called Health Behaviour in School-aged Children, or HBSC (Craig et al., 2020). Affiliated with WHO-Europe and an international survey network (Inchley et al., 2020), HBSC involves a periodic survey of nationally representative samples of 11- to 15-year-old children in 50 countries, including Canada. The survey questionnaire includes items and scales describing many aspects of mental health as well as contextual factors, sociodemographic features and health risk behaviours that act as possible determinants. HBSC has been conducted on eight occasions in Canada since 1990; Cycle 8 (2017-18) included survey reports from 21,587 young people from 287 Canadian schools (Craig et al., 2020).

The primary study sample included adolescents who participated in the 2017-18 Canadian HBSC study. This sample was designed to be nationally representative by grade and sex within Canada as a whole, and each of the 12 provinces and Territories (Nunavut did not participate). Inclusion criteria were: (1) attending any of the 287 schools selected for study in the 12 jurisdictions; (2) provision of consent as per local school board customs; and (3) completion of required survey items. There were no specific exclusions.

### 2.2 Variables for study

HBSC contains a large number of standard questionnaire items focusing on the lives and health behaviours of adolescents, and, following extensive piloting, expressed in a manner and at a literacy level that is appropriate for children as young as 11 years. The origins and validity of these measures are documented in an international protocol (Currie et al., 2014). With standard terms used in the field of intersectionality, they include items that can be combined and analyzed as *social locations*, as well as various indicators of mental health status.

#### 2.2.1 Social location variables

*Gender.* In the HBSC, participants report whether they are “female” or “male”, or “neither term describes me”^1^. Due to small sample sizes, those who indicated that “neither term describes me” were, of necessity, excluded.

*Grade.* Grade level (range 5–11) is indicated. Because grade is highly correlated with age, it is the primary indicator used for a child's developmental stage.

*Urban-rural status.* Based on the school address and postal code, Statistics Canada urban-rural coding systems (Statistics Canada, 2017a, Statistics Canada, 2017b) are used to infer urban-rural geographic status in four categories: rural area (<1000 population), and small (1000 to 29,999), medium (30,000 to 99,999) and large (100,000+) population centres.

*Family Socio-economic Status.* Individual family affluence (FAS III; the validated HBSC measure of socioeconomic status) (Hartley et al., 2016) is measured by assessing participants’ answers to six items describing the material conditions of their household. Responses to individual items are summed into a scale.

*Relative material wealth.* A single item asks young people to rate “how well off do you think your family is?”, with five response options ranging from “very well off” to “not at all well off” (Currie et al., 2008).

*Family structure.* For the home that they live in most often, participants indicate whom they live with (father and mother, mother and partner, mother only, father and partner, father only, other - including foster home).

*Immigration status.* A single item is used to describe where participants were born (e.g., “Canada”, “other country” [with specification], or “unknown”). Another item indicates the number of years that they have lived in Canada (born in Canada, Immigrated <5 years ago, Immigrated 5 or more years ago).

*Religious Involvement.* As part of a simple scale to gauge involvement in organized activities or groups, participants are asked (yes or no) whether they are involved in a church or other religious/spiritual group (Badura et al., 2021).

*Racial or Cultural Background.* Participants indicate their identity from a standardized list of categories that combine race and ethnicity, based on a Statistics Canada protocol (Statistics Canada, 2017a, Statistics Canada, 2017b).

#### 2.2.2 Indicators of mental health

*Positive Psychosomatic Health.* The HBSC subjective health complaints index (Hetland et al., 2002) was used as an indicator of positive mental health. This index asks about the frequency of somatic and psychological symptoms that may impair everyday function. In the checklist youth report how often in the last 6 months (0 = “rarely or never” to 4 = “about every day”) they experienced the following: headache, stomach-ache, backache, feeling depressed or low, irritability or bad temper, feeling nervous, difficulties in getting to sleep, and feeling dizzy (Cronbach's α = 0.84) (Hetland et al., 2002). Responses were summed to create a composite scale ranging from 0 to 32. Scores were reverse coded so that higher scores indicate more positive mental health.

*Depression.* For the past 12 months, participants report whether they “ever felt so sad or hopeless almost every day for two weeks or more in a row that they stopped doing some usual activities” (yes or no) (Hallfors et al., 2004).

*Diagnoses.* As part of a brief scale describing documented learning exceptionalities at school, participants indicate (yes or no) whether they have a diagnosed mental illness (e.g., depression, anxiety, bipolar disorder).

### 2.3 SIDES analysis

Our primary analysis involved a recursive partitioning technique titled *Subgroup Identification based on Differential Effect Search (SIDES)* (Lipkovich et al., 2011). This algorithm identifies “subgroups for which there is a large treatment effect (treatment by subgroup interaction)” (Mistry et al., 2018). When applied to our study of differences in mental health between males and females (with “males” or “females” as our hypothetical “treatment”), it identified subgroups of the population where differences in mental health between males and females were particularly pronounced. SIDES was designed specifically for studying treatment-subgroup interactions for a variety of outcome types (Doove et al., 2014) and can be implemented using recently-developed, freely available software (Riviere, 2019). SIDES was chosen instead of other recursive partitioning methods because, rather than focusing on finding a single best partition of the entire covariate space, it is designed to identify multiple (possibly overlapping) subgroups with large treatment effects (Doove et al., 2014). This is particularly desirable in our setting because we want to identify any (possibly overlapping) intersections of social locations where inequalities in mental health between males and females were particularly pronounced.

For a given covariate (social location variable), the SIDES algorithm considers all possible binary splits of the data, and uses a pre-determined *splitting criterion* to identify the optimal splitting point that maximizes the differences in mental health outcomes between males and females in one of the two produced subgroups. In other words, the algorithm identifies where the data can be best split (into subgroups 1 and 2) based on this covariate in order to minimize

(1) $$p_{split}=2\;\cdot \min\left\{1-\text{Φ}\left(Z_{1}\right),1-\text{Φ}\left(Z_{2}\right)\right\},$$

where $Z_{S}$ is a test statistic for testing whether males and females in subgroup S differ significantly in terms of their mean mental health outcome, and Φ is the CDF of a standard normal distribution (Lipkovich et al., 2011). For example, using the *Grade* covariate, we would consider all possible binary splits of the data (Grade 5 vs Grade ≥6; Grade <7 vs Grade ≥7; Grade <8 vs Grade ≥ 8; etc.) and look for the split that produced a subgroup where males and females differed most in terms of, for example, the rate of diagnosed mental illnesses. Each covariate is considered in turn and ranked based on the size of the difference in mean mental health outcome between males and females in the subgroup produced by the optimal split for that covariate (SIDES ranks the covariates in terms of their optimal splitting criterion p-value, after applying a multiplicity adjustment to avoid bias toward selecting covariates with greater numbers of potential splits).

Data that were being split were referred to as *parent nodes*, and the groups that resulted from these splits were *child nodes*. Child nodes resulting from the optimal splits of the best *M* = 5 covariates were considered further, and those with the largest differences were deemed to be a *promising subgroup*, provided that they satisfied predetermined *continuation* or *complexity* criterion that require a child node to offer significant improvement over its parent node. We then used the algorithm to recurse within each promising subgroup to explore further subdivisions; this process was capped so that subgroups were defined by intersections of at most *L* = 3 covariates. Finally, a resampling procedure was employed to compute an adjusted p-value for each promising subgroup to reduce the likelihood of false positives (Lipkovich et al., 2011; Mistry et al., 2018).

In our setting, we encountered difficulties when applying the original SIDES algorithm due to our large sample size and large overall treatment effect (the large overall inequality between males and females in terms of their mental health outcomes resulted in extremely large values of $Z_{S}$ and correspondingly tiny p-values that were subject to problematic roundoff error). To overcome similar challenges, Mistry et al. (Mistry et al., 2018) applied an alternate splitting criterion that considered a two-sided test for “the differential effect of two nodes” rather than identifying the single subgroup with the most significant treatment effect. Given our desire to identify subgroups demonstrating an increased inequality between mental health outcomes between males and females, we implemented a different modification to the SIDES algorithm; we defined the "minimum desired difference to be demonstrated between the treatment and the control” (the parameter *D*, in the SIDES algorithm implemented in *R* (Riviere, 2019)) to be the inequality in mental health outcome observed between males and females in the whole sample. In other words, if *X* represents a continuous measure of mental health outcome, then in (eq:#1), we used

(2) $$Z_{S}=\frac{\left(\;{\bar{\text{X}}}_{female,\;S}-\;{\bar{\text{X}}}_{male,\;S}\right)-\left(\;{\bar{\text{X}}}_{female,\;overall}-\;{\bar{\text{X}}}_{male,\;overall}\right)}{SE}$$

where $\;{\bar{\text{X}}}_{female,\;S}$ is the mean of the health outcome observed for females in subgroup *S*, $\;{\bar{\text{X}}}_{female,\;overall}$ is the mean of the health outcome observed for females in the entire sample, and SE is the standard error of the estimator $\;{\bar{\text{X}}}_{female,\;S}-\;{\bar{\text{X}}}_{male,\;S}.$ By centering our subgroup test statistics in this way, our implementation of SIDES avoids roundoff error due to overly large values of $Z_{S}$ and directly tests whether a candidate subgroup represents a subpopulation with a larger inequality between males' and females' mental health than exists in the overall sample.

## 3 Results

Table 1 describes the available study population, stratified by gender. HBSC provided robust and diverse samples by gender, grade level, and urban-rural geographic status. The majority of participants lived in homes with two adult partners (78.1% of males, 78.0% of females), reported “white” as a racial or cultural background (66.6% of males, 66.1% of females), were born in Canada (74.4% of males, 78.4% of females), and were classified as having “average” or “high” relative levels of wealth (91.5% of males, 90.8% of females). A minority reported belonging to a church or other religious/spiritual group (24.1% of males, 25.7% of females).

**Table 1:** Description of the 2018 Canadian HBSC sample of males and females aged 11–15 years.

Variable	Males (n = 10,349)	Females (n = 10,872)
**Grade**		
5 and 6	2037 (19.7%)	1949 (17.9%)
7	2143 (20.7%)	2315 (21.3%)
8	2114 (20.4%)	2264 (20.8%)
9	2295 (22.2%)	2415 (22.2%)
10 and 11	1759 (17.0%)	1929 (17.7%)
Missing	1	0
**Urban/rural status**		
Rural area (<1000)	442 (4.3%)	440 (4.0%)
Small population center (1000 to 29,999)	5439 (52.6%)	5562 (51.2%)
Medium population center (30,000 to 99,999)	1809 (17.5%)	1887 (17.4%)
Large population center	2659 (25.7%)	2983 (27.4%)
Missing	0	0
**Family SES (based on having computer, bedroom, bathroom, car, dishwasher, holiday)**		
Quintile 1	1436 (17.5%)	1735 (19.0%)
Quintile 2	1701 (20.7%)	1876 (20.6%)
Quintile 3	1641 (20.0%)	1772 (19.4%)
Quintile 4	1643 (20.0%)	1798 (19.7%)
Quintile 5	1790 (21.8%)	1942 (21.3%)
Missing	2138	1749
**Family structure**		
Mother and father	7279 (72.3%)	7525 (70.6%)
Mother and partner	481 (4.8%)	656 (6.2%)
Mother only	1545 (15.3%)	1701 (16.0%)
Father and partner	103 (1.0%)	141 (1.3%)
Father only	323 (3.2%)	303 (2.8%)
Other (including children's or foster home)	341 (3.4%)	338 (3.2%)
Missing	277	208
**Immigration status**		
Born in Canada	7218 (74.4%)	7948 (78.4%)
Immigrated less than 5 years ago	697 (7.2%)	683 (6.7%)
Immigrated more than 5 years ago	1785 (18.4%)	1513 (14.9%)
Missing	649	728
**Affluence**		
High	4932 (55.7%)	4912 (51.2%)
Average	3174 (35.8%)	3805 (39.6%)
Low	749 (8.5%)	880 (9.2%)
Missing	1494	1275
**Religious**		
Yes	2359 (24.1%)	2693 (25.7%)
No	7432 (75.9%)	7766 (74.3%)
Missing	558	413
**Ethnicity**		
White	6770 (66.6%)	7066 (66.1%)
Black	369 (3.6%)	369 (3.4%)
Indigenous	793 (7.8%)	819 (7.7%)
East Asian	609 (6.0%)	626 (5.9%)
West Asian and Arab	146 (1.4%)	145 (1.4%)
Other	1480 (14.6%)	1671 (15.6%)
Missing	182	176

**Fig. 1:**
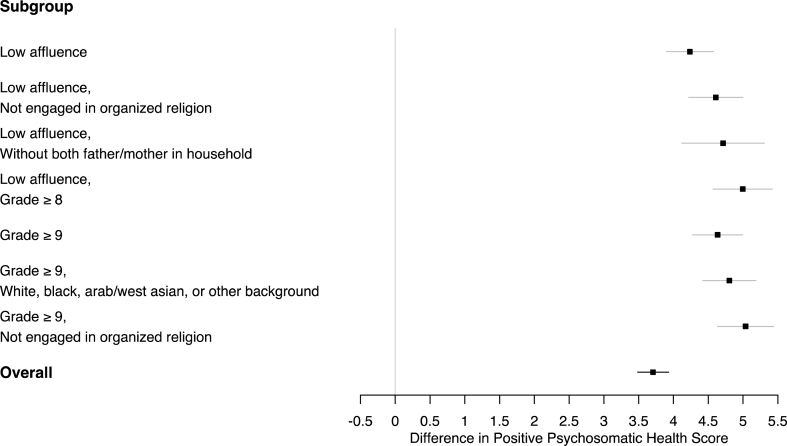
Subgroups of the population where the difference between males' and females' average positive psychosomatic health scores was significantly pronounced; overall, males scored approximately 3.7 points higher on this 32-point scale relative to females.

In Fig. 1, we present subpopulations where differences between adolescent males' and females' average *positive psychosomatic health* scores were significantly pronounced. Overall, males scored approximately 3.7 points higher on average on this 32-point scale relative to females (a 95% confidence interval for the difference was 3.48–3.92). This inequality between adolescent males and females was significantly more pronounced in subpopulations defined by intersections of low affluence, upper-level grades, non-engagement in organized religion, households without both the father and the mother, and those who reported a racial or cultural background other than Indigenous or East Asian. In particular, the inequality between males' and females’ average Positive Psychosomatic Health Score was largest among adolescents of low affluence in upper-level grades (95% CI of 4.57–5.42) and among adolescents in upper-level grades who were not engaged in organized religion (95% CI of 4.63–5.44).

**Fig. 2:**
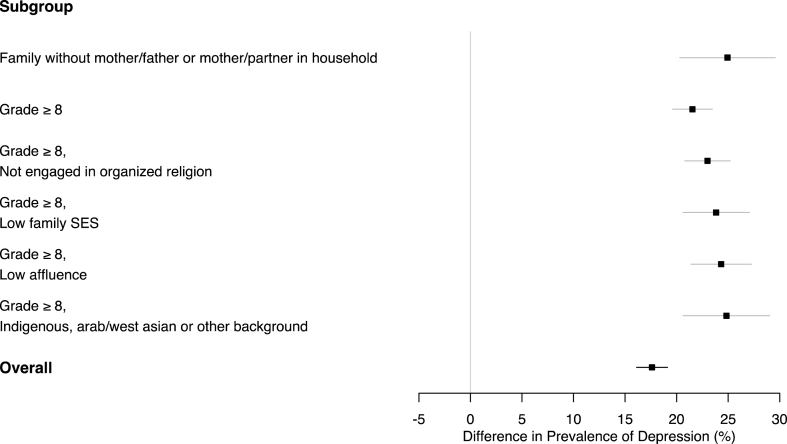
Subgroups of the population where the difference in prevalence of symptoms of depression between males' and females' was significantly pronounced; overall, the prevalence of symptoms was much more common among females (40.1%) compared with males (22.5%).

Fig. 2 presents subpopulations displaying significant differences between adolescent males and females in terms of the first of two negative mental health outcomes: *symptoms of depression*. Overall, the prevalence of symptoms was much more common among females (40.1%) compared with males (22.5%) (a 95% CI for the inequality in prevalence was 16.1%–19.1%). This difference between males and females was again significantly more pronounced in subpopulations defined by intersections of low affluence (or low family SES), upper-level grades, non-engagement in organized religion, and, in this setting, households without both the father and the mother or without the mother and partner, and those who reported a racial or cultural background other than White, Black, or East Asian. For example, the prevalence of symptoms of depression was 24.3% higher in females than in males when considering adolescents of low affluence in upper-level grades (95% CI of 21.4%–27.3%).

**Fig. 3:**
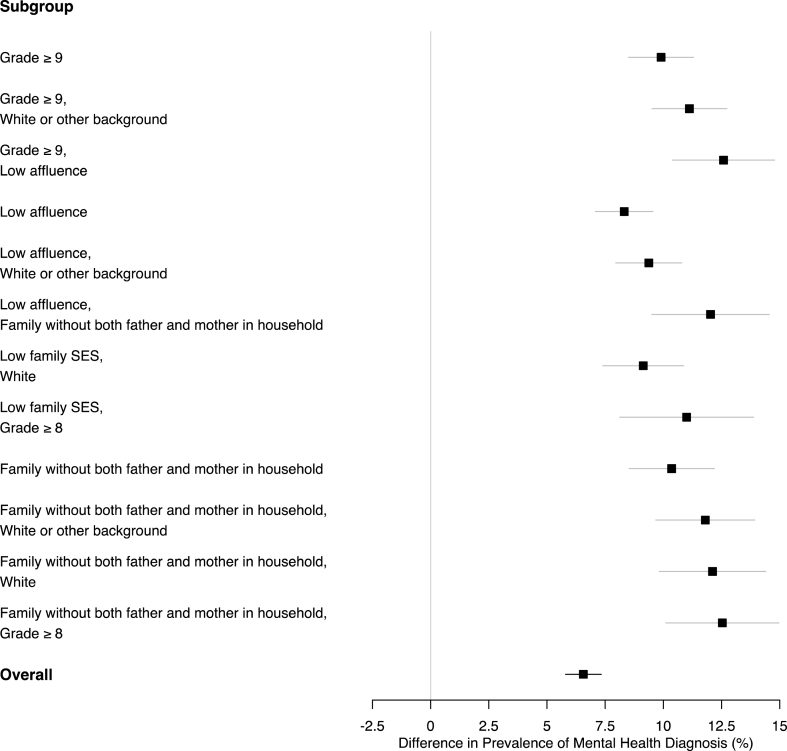
Subgroups of the population where the difference in prevalence of mental health diagnoses between males' and females' was significantly pronounced; overall, this outcome was reported by 2.8% of males and 9.3% of females.

Fig. 3 presents subpopulations displaying significant differences between adolescent males and females for the second negative mental health outcome: *presence of a diagnosed mental health illness*. This outcome was reported by 2.8% of males and 9.3% of females (a 95% CI for the inequality in prevalence was 5.8%–7.3%). The gap between males and females was again significantly more pronounced in subpopulations defined by intersections of low affluence (or low family SES), upper-level grades, households without both the father and the mother, and those who reported specific racial or cultural backgrounds (here, primarily backgrounds other than Black, Indigenous, East Asian, West Asian and Arab). In particular, the difference between males' and females’ prevalence of a diagnosed mental health illness was largest among adolescents of low affluence in upper-level grades (95% CI of 10.4%–14.8%) and among adolescents in upper-level grades who live in households without both their father and mother (95% CI of 10.1%–15.0%).

Table 2 displays the mental health outcomes (and corresponding 95% confidence intervals) of adolescent males and females overall and in the intersection that was identified as being important for all three outcomes: older adolescents of low affluence. Prevalence of mental health diagnoses among males was 2.8% overall, but 4.0% among older males of low affluence. At the same time, mental health diagnoses among females was 9.3% overall, but 16.5% among older females of low affluence. While overall, the difference between adolescent males' and females’ rate of diagnoses of mental health problems was 6.6%, this almost doubled to 12.6% among older adolescents of low affluence. Similarly, the prevalence of symptoms of depression was higher for older adolescent males of low affluence (29.4%) than for adolescent males overall (22.5%), but the span was more pronounced for females (53.7% among older adolescent females of low affluence compared to 40.1% overall). Therefore, the difference between males and females in terms of the prevalence of symptoms of depression was significantly more pronounced among older adolescents of low affluence (24.3% compared to 17.6% overall). Finally, when considering only older adolescents of low affluence, both males and females had lower average positive psychosomatic health scores than did their overall cohorts (31.6 vs 32.9 for males and 26.6 vs 29.2 for females). This means that the difference between males and females in terms of the average positive psychosomatic health scores was significantly more pronounced among older adolescents of low affluence (5.0 compared to 3.7 overall; see Table 2 for the corresponding confidence intervals).

**Table 2:** Mental health outcomes (and corresponding 95% confidence intervals) of males and females overall and in the intersection that was identified as being important for all three outcomes: older adolescents of low affluence.

	Average positive psychosomatic health score			Prevalence of symptoms of depression (%)			Prevalence of a diagnosed mental health illness (%)		
	Males	Females	Difference	Males	Females	Difference	Males	Females	Difference
Overall	32.9 (32.8, 33.1)	29.2 (29.1, 29.4)	3.7 (3.5, 3.9)	22.5 (21.5, 23.5)	40.1 (39.0, 41.2)	17.6 (16.1, 19.1)	2.8 (2.4, 3.2)	9.3 (8.7, 10.0)	6.6 (5.8, 7.3)
Older adolescents of low affluence	31.6 (31.3, 31.9)	26.6 (26.3, 26.9)	5.0 (4.5, 5.4)	29.4 (27.3, 31.5)	53.7 (51.6, 55.7)	24.3 (21.4, 27.3)	4.0 (3.0, 5.2)	16.5 (14.8, 18.5)	12.6 (10.4, 14.8)

## 4 Discussion

Adolescent females generally report poorer mental health than do adolescent males. In particular, in the HBSC data, females have significantly lower positive psychosomatic health score and significantly higher prevalence of depression and diagnosed mental health illnesses*.* We used a recursive partitioning approach to identify subpopulations (defined by upwards of three-way interactions of social locations) where the inequality between males and females was even more pronounced than in the overall population. We hoped to identify social locations that can then be studied further to determine whether or not the poorer outcomes experienced by subpopulations identified by our analysis can be attributed to the intersection of gender with the social and environmental factors we identified.

We found that mental health gaps between males and females were significantly more pronounced in subpopulations defined by intersections of older age, low affluence (or low family SES), non-engagement in organized religion, households without both father and mother, and among those of specific racial or cultural backgrounds. For example, the difference between males' and females’ average mental health outcomes was generally significantly more pronounced among older adolescents, among those of low affluence, and, in particular, among those who were both older and of low affluence (i.e., within the intersection of older age and low affluence).

Our analyses used the Subgroup Identification based on Differential Effect Search (SIDES) algorithm for recursive partitioning (Lipkovich et al., 2011; Mistry et al., 2018). Unlike traditional recursive partitioning methods that partition data into subgroups that are relatively homogenous in terms of a particular outcome variable (Zhang & Singer, 2013), the SIDES method searches for specific subpopulations where the outcome differs most significantly across levels of a predictor of interest, in our case males and females.

When viewed through the lens of intersectional theory (Crenshaw, 1991, pp. 1241–1299; Bauer, 2014; Bauer & Scheim, 2019), these quantitative, data-driven findings suggest that unequal mental health experiences of adolescent females and males may have been driven by the circumstances experienced by the specific subpopulations to which each participant belonged. Indeed, we saw considerable promise in our approach and findings. Social locations that have traditionally been associated with intersectionality in adult populations (e.g., sex/gender intersecting with racial or cultural identify, and economic deprivation, to produce health advantages and disadvantages) (Crenshaw, 1991, pp. 1241–1299) did emerge here, but for outcomes (mental health) and in a population (adolescents) where this theory has rarely been applied and tested. This alignment with existing literature on adults adds credibility to our findings with respect to adolescents. Additionally, the SIDES algorithm identified several other largely unexplored facets of social locations with gender and health (e.g., grade level/age, households without both the father and the mother, and religious involvement) that may shape mental health of adolescent populations.

The value of this analysis lies not only in our ability to quantify differences in mental health status between Canadian adolescent males and females, but also in our demonstration of the utility of this statistical method to identify and quantify such inequalities. We view this as a first step to support causal mediation analyses exploring deeper explanations for how and why such inequalities arise (VanderWeele et al., 2012). By intentionally focusing on these phenomena during childhood, we hope to understand more about the origins of health inequalities that persist into adulthood, and thereby more fully inform social and other types of policy. Strengths of this analysis therefore arise from its novelty to the adolescent health and intersectionality literatures, our adaptation of the SIDES algorithm to an analysis comparing mental health for adolescent males and females, and our identification of established and new social locations for in-depth study.

Our study has important limitations. The cross-sectional nature of HBSC precludes the possibility of establishing the direction of relationships, although many of the factors contributing to social locations are invariant and unlikely to be affected by mental health status. Analyses are based upon self-reports from children as young as 11 years, and misclassification of key variables and bias to effects are likely. Finally, analysis was also limited to available factors documented in a pre-existing dataset. Additional social locations of importance remain possible.

While our analysis was able to provide evidence of subpopulations representing possible social locations where mental health differences between adolescent males and females exist, it provides no evidence about the mechanisms that underlie such differences. As discussed above, this requires more in-depth study to identify how and why such differences emerge between adolescent males and females in Canada. A common hypothesis in intersectional thinking is that when multiple disadvantages (or privileges) are experienced simultaneously, the resultant health advantages or disadvantages are more than, and different from, additive effects of each (Bauer, 2014). Possible explanations for the overall gap in mental health between adolescent males and females include differences in the expectations put on males' and females’ behaviours, the acceptability of acknowledging and reporting mental health and illness (Kroenke & Spitzer, 1998), variations in risk-taking, substance use and other behaviours (Kloos et al., 2009), and differential experiences with social media use (Boer et al., 2020). Such hypotheses should be tested both qualitatively and quantitatively to further understand the social roots of mental health experiences among Canadian youth.

### 4.1 Conclusions

In summary, this original analysis used a novel application of a data mining technique to identify subpopulations, defined by intersecting social locations, where the mental health of adolescent males and females differs most substantially. We found mental health differences between adolescent males and females to be significantly more pronounced in subpopulations defined by intersections of gender with low affluence (or low family SES), upper-level grades, non-engagement in organized religion, households without both the father and the mother, and among those of specific racial or cultural backgrounds. We view this as foundational research aimed at understanding the origins of at least some gender-based health inequalities at a critical stage of life. Increased intersectional understanding about mental health such as we have demonstrated in this analysis could provide a stronger empirical foundation for monitoring trends, making policy-decisions and supporting mental health of both adolescent males and females. This better understanding of health inequalities can thus lead to determined and focused action to address health inequities.

## Funding

The current research was done in conjunction with a 10.13039/501100000024Canadian Institutes of Health Research Grant (grant #DC0190GP) held by the primary authors. The HBSC study is funded in Canada by the 10.13039/100011094Public Health Agency of Canada. Principal Investigators are Dr. Wendy Craig (Queen's University) and William Pickett (Brock and Queen's Universities), and its national coordinator is Mr. Matthew King (Queen's University). The funding sources had no involvement in the study design; in the collection, analysis and interpretation of data; in the writing of the report; or in the decision to submit the article for publication.

## Author statement

**MAMcIsaac**: Conceptualization, Methodology, Writing - Original Draft, Funding acquisition.

**M Reaume**: Methodology, Software, Visualization.

**SP Phillips**: Writing - Original Draft, Funding acquisition.

**V Michaelson**: Writing - Review & Editing, Funding acquisition.

**V Steeves**: Writing - Review & Editing, Funding acquisition.

**CM Davison**: Writing - Review & Editing, Funding acquisition.

**A Vafaei**: Writing - Review & Editing.

**N King**: Writing - Review & Editing.

**W Pickett**: Conceptualization, Investigation, Writing - Original Draft, Funding acquisition.

## Ethical statement

Ethics approval for the Health Behaviour in School Aged Children Survey was sought and obtained from Health Canada, the Public Health Agency of Canada, and the General Research Ethics Board at Queen's University. Following receipt of permission from the 10 provinces and 3 territories in Canada, active consent was provided by school boards and individual schools, while passive consent was obtained from student participants and their parents or guardians.

## Declaration of competing interest

None.

## Footnotes

1 We understand gender to describe a broad spectrum of identities and that asking about being male or female is not the same as asking about gender identity. Nonetheless, our hope in applying SIDES, in a proof-of-concept activity to the current study dataset including this existing question, is to test a process.
